# Acute blood biomarker responses to consensual sexual choking/strangulation in young adult women: a randomized crossover study

**DOI:** 10.3389/fgwh.2025.1717361

**Published:** 2026-01-08

**Authors:** Sage H. Sweeney, Grace O. Recht, Giselle Lima-Cooper, Dibyadyuti Datta, Claire V. Buddenbaum, Harper Day, Bella Buehler, Megan E. Huibregtse, Debby Herbenick, Keisuke Kawata

**Affiliations:** 1 Department of Kinesiology, Indiana University School of Public Health—Bloomington, Bloomington, IN, United States; 2 Department of Pediatrics, Indiana University School of Medicine, Indianapolis, IN, United States; 3 Department of Psychiatry and Behavioral Sciences, Emory University School of Medicine, Atlanta, GA, United States; 4 Department of Health and Kinesiology, University of Illinois Urbana-Champaign, Urbana, IL, United States; 5 Department of Applied Health Science, Indiana University School of Public Health-Bloomington, Bloomington, IN, United States; 6 The Center for Sexual Health Promotion, Indiana University School of Public Health-Bloomington, Bloomington, IN, United States; 7 Program in Neuroscience, College of Arts and Sciences, Indiana University, Bloomington, IN, United States

**Keywords:** blood biomarker, brain health, choking, neuroinflammation, sexual behaviors, sexual strangulation, intimate partner violence

## Abstract

**Introduction:**

Sexual strangulation, commonly referred to as “choking”, has become increasingly common among young adults, yet its neurobiological consequences remain poorly understood. Preclinical and clinical evidence suggests strangulation may trigger axonal injury, neuroinflammation, and blood–brain barrier dysfunction. Blood biomarkers of neural injury and inflammation provide a sensitive means to detect subtle effects.

**Aim:**

To examine whether consensual sexual choking/strangulation acutely alters blood biomarkers of neural injury and inflammation compared to non-choking sexual activity in young adult women.

**Methods:**

In a randomized crossover study, 29 women (mean age 21.5 ± 2.7) completed three laboratory visits: baseline (≥24 h abstinence), post-choking sex, and post-non-choking sex. Blood was collected within 24 h of sexual events. Neural injury biomarkers (NfL, tau, GFAP, UCH-L1, S100B) and inflammatory markers (IL-1ra, TNF-R1, CCL-2, VEGF-A, VCAM-1) were analyzed using Quanterix and Luminex multiplex immunoassays. Mixed-effects regression models tested exposure-by-time interactions, adjusting for age and brain trauma history.

**Results:**

Neurofilament light (NfL) significantly increased after choking-involved sex but remained unchanged after non-choking, which resulted in a statistically significant exposure-by-time interaction [*β* = −0.21, 95% CI (−0.38, −0.03), *p* = 0.021]. Other neural biomarkers did not differ by exposure. Among inflammatory markers, CCL-2 and VEGF-A demonstrated a similar pattern as NfL, with acute increases after choking-involved sex, but not following non-choking sex, yielding in exposure-by-time interaction effects (CCL-2: *β* = −14.60, 95% CI [−25.70, −3.43, *p* = 0.011; VEGF-A: *β* = −9.29 (−19.71, 1.13), *p* = 0.079]. IL-1ra, TNF-R1, and VCAM-1 remained stable.

**Discussion:**

Consensual sexual strangulation elicited acute increases in NfL and CCL-2, with VEGF-A showing a similar pattern, suggesting transient axonal stress and hypoxia-related inflammatory signaling. These findings indicate that sexual choking/strangulation, even in consensual contexts, may have subtle, yet detectable cellular burden. Future studies with larger samples, refined temporal sampling, and multimodal outcomes are needed to clarify short- and long-term implications.

## Introduction

In recent years, sexual strangulation–commonly referred to as “choking”–has become increasingly prevalent among young adults (1). In the United States (U.S.), nationally representative surveys indicate that over 30% of women aged 18–29 report having been choked/strangled by a partner during consensual sex (2). Further, data from randomly sampled college students show that 37% of women who reported such experiences have engaged in this behavior on more than five occasions (3). While often described as consensual and arousing, this behavior involves neck compression that can restrict cerebral blood flow, airflow, or both (1, 4). Emerging clinical evidence suggests associations between exposure to sexual choking/strangulation and functional impairments, including difficulty breathing, swallowing, and speaking (3). About one in five women who have been choked during sex report having experienced at least one alteration in consciousness such as blurred vision, disorientation, dizziness or loss of consciousness (3). Furthermore, women who report experiencing sexual choking/strangulation five or more times in their lifetime are twice as likely to report symptoms of depression, loneliness, sadness, and anxiety compared to choking-naïve individuals (5). These psychological symptoms may signal cellular stress, highlighting the need to investigate underlying neurobiological mechanisms.

Moreover, sexual choking/strangulation is not always consensual (or meaningfully consensual), with studies in the U.S. and Sweden showing that non-fatal strangulation is increasingly reported as part of sexual assaults among young women (6–8). Some women who consent to being choked do so because they perceive it as normative, feel it is expected during sex, or wish to please their partner (4). A nationally representative U.S. survey data, college campus-representative surveys, and qualitative interviews further demonstrate that some women find the experience as scary, especially when it occurs without prior discussion or when the intensity exceeds expectations (4, 9, 10). It is also important to acknowledge that many individuals engage in sexual choking/strangulation for reasons related to enhancement of pleasure, erotic intensity, or connections. Prior research demonstrates that choking/strangulation may increase sexual arousal or intimacy for some individuals, particularly when it occurs under consensual activities (4, 9, 11). Within the DSM-5 framework, such behaviors can be considered within the spectrum of paraphilias, which are not considered pathological, unless they meet criteria for paraphilic disorders that can trigger impaired judgement involving non-consenting partners (12, 13). It should be recognized that sexual choking/strangulation exists across diverse motivational contexts, which underscore the critical need for research on sexual choking/strangulation and its potential health consequences in both consensual and non-consensual sex.

From a pathophysiological standpoint, even brief episodes of cerebral hypoperfusion or hypoxia, both of which can result from strangulation, can disrupt brain homeostasis. Evidence from preclinical studies and other clinical contexts (e.g., autoerotic asphyxiation or the “choking game”) indicates that strangulation may induce neuroinflammation, trigger neuronal damage, and compromise blood–brain barrier (BBB) integrity (14, 15). In non-consensual, violent contexts, such as intimate partner violence (IPV), these effects have been associated with cognitive dysfunction, chronic headache, depression, and structural brain changes (16–19). Extending this literature, our recent work among young adult females with frequent sexual strangulation exposure revealed cortical thickening in frontoparietal regions (20), interhemispheric imbalance in resting-state neural activity (21), and compensatory recruitment of cognitive networks during memory tasks (22). These data suggest that sexual choking/strangulation may pose threats to brain health.

Blood-based biomarkers offer a sensitive, noninvasive means of detecting the biological aftermath of brain stress. Proteins such as neurofilament light (NfL), tubulin-associated unit (tau), glial fibrillary acidic protein (GFAP), ubiquitin C-terminal hydrolase L1 (UCH-L1), and S100 calcium-binding protein beta (S100B) are released into circulation following various forms of neural injury (23, 24). NfL and tau are cytoskeletal proteins abundant in myelinated axons and released upon axonal disruption or cytoskeletal destabilization (25), with elevated levels observed even in subclinical or subconcussive head impacts (26, 27). GFAP is an intermediate filament protein localized to astrocytes, supports blood–brain barrier (BBB) integrity, and modulates cerebral inflammation. Its release reflects astrocyte activation or injury (23, 24). Another astrocyte-enriched marker, S100B, is implicated in neuroinflammation and has showed to elevate in young adult women recently and frequently exposed to partnered sexual choking/strangulation (28). UCH-L1, a neuron-specific enzyme involved in protein degradation and repair, reflects neuronal cell body damage. These biomarkers have been validated across diverse neurological conditions, including sports-related concussion, blast injury, stroke, and Alzheimer's disease (23, 29, 30), and exhibit clear temporal dynamics following injury, making them well-suited for detecting acute neurological stress.

Meanwhile, circulating cytokines and vascular markers provide insight into systemic and neuroimmune responses following hypoxia and ischemia (31). Tumor Necrosis Factor-Alpha (TNF-α) amplifies oxidative stress and neurovascular dysfunction through binding to its receptor (TNF-R1), while Interleukin-1 receptor antagonist (IL-1ra) serves as a counter-regulatory anti-inflammatory signal. Vascular Cell Adhesion Molecule-1 (VCAM-1), upregulated during inflammation, facilitates leukocyte migration across the BBB and reflects microvascular compromise (32). CCL-2 and VEGF-A, markers of chemotaxis and angiogenesis, are responsive to hypoxia and mediate BBB disruption and edema formation (33). Recent data from Sun et al. (34) highlight the relevance of these pathways in IPV-related brain injury. Specifically, their novel rodent strangulation model that restricts the airway for 90 s revealed increased expression of proinflammatory genes, including *Ccl-2*, *Il-1α*, and *Vegf-a*, in the prefrontal cortex, temporal cortex, and hippocampus, despite the absence of overt structural damage (34). These data support the utility of inflammatory biomarkers in detecting diffuse neural injury including the acute effects of sexual choking/strangulation.

Therefore, we conducted a randomized crossover study to examine plasma levels of five brain injury biomarkers (NfL, tau, GFAP, UCH-L1, S100B) and a panel of inflammatory markers (including IL-1ra, TNF-R1, CCL-2, VEGF-A, and VCAM-1) within 24 h following consensual sexual events involving choking/strangulation compared to non-choking/strangulation sex. We hypothesized that acute experience of sexual choking/strangulation would be associated with elevated levels of brain-derived and inflammatory biomarkers, indicative of transient neural stress or vascular dysfunction, whereas biomarker levels would remain consistent after non-choking/strangulation events.

## Materials and methods

### Participants

**Figure 1:**
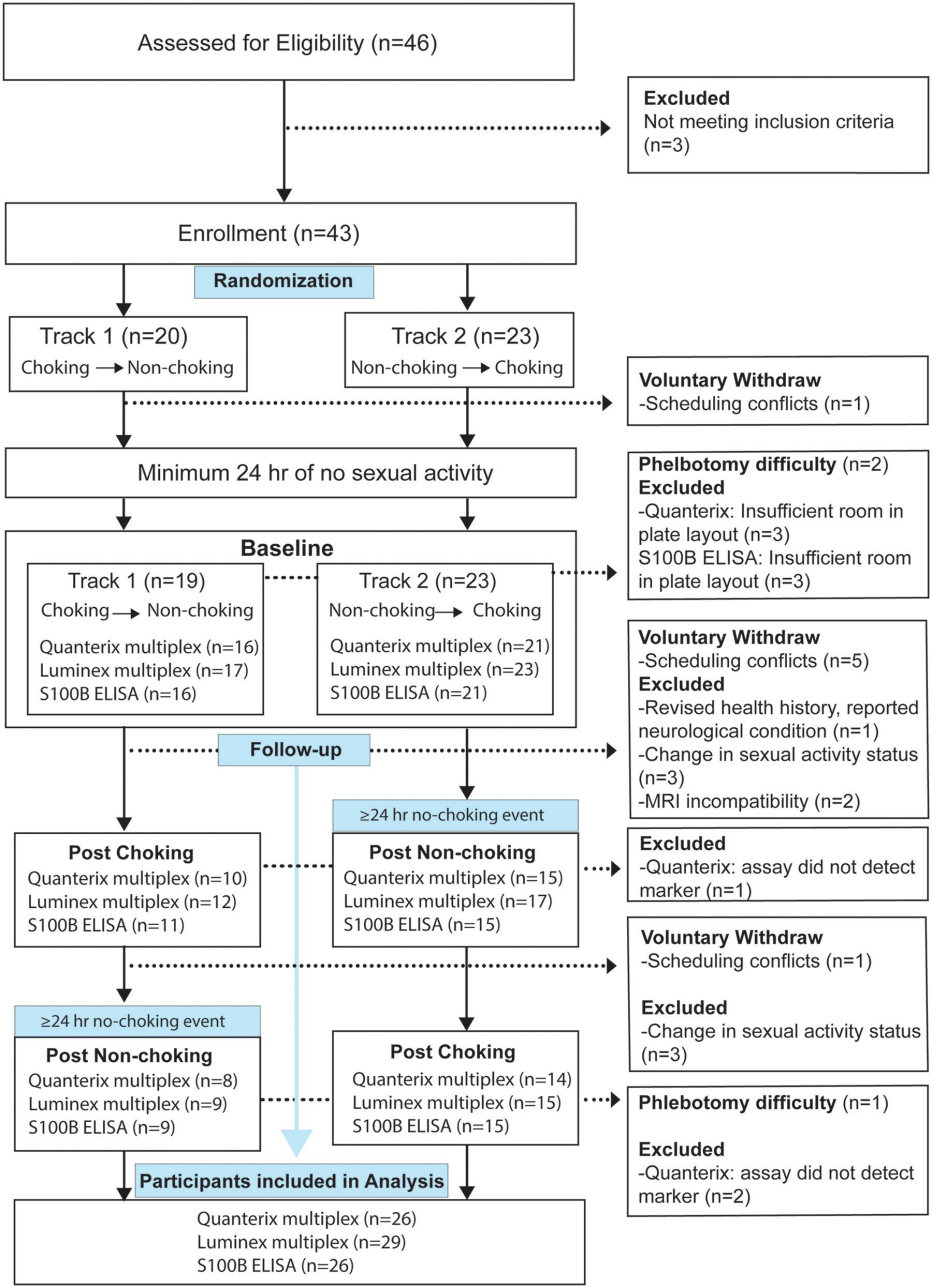
Study flow chart.

This randomized crossover study included 43 female participants aged 18–30 years who were recruited through Indiana University (IU) Classifieds advertisements, social media posts, Indiana Clinical and Translational Sciences Institute (CTSI)'s iConnect, and IU Canvas course pages. Data were collected from October 2024 to April 2025. Inclusion criteria were assigned female at birth, English fluency, and engagement in partnered sex involving choking/strangulation on at least two occasions in the past month. The criterion of at least two choking/strangulation events in the past month ensured that participants were likely to experience both exposure conditions (choking and non-choking sex) within the study window, which was essential for the randomized crossover design. Exclusion criteria included being currently pregnant, a history of neurological disorders (e.g., seizures, brain tumors), psychotic symptoms, antipsychotic medication use, fewer than two choking events in the past month, or exclusive engagement in choking-involved sex. Study flow is depicted in Figure 1. All participants provided informed consent prior to the start of any study procedures. The study protocol was approved by the Indiana University Institutional Review Board (#23297). The protocol was also registered under Clinicaltrials.gov (NCT06602362).

#### Study procedures

To reduce expectancy biases and limit behavioral changes, participants were not informed that the study specifically examined sexual choking/strangulation. Instead, they were told the research focused on how various emerging sexual behaviors affect the brain. Because the true purpose of the study was concealed to minimize expectancy effects, participants were not explicitly informed that choking/strangulation was the focal exposure. Each participant attended three sessions: one baseline visit conducted after at least 24 h of sexual abstinence, and two follow-up sessions: one within 24 h after engaging in sex that involved choking/strangulation, and one within 24 h after sex without choking. The sequence of follow-up visits was randomized using R's random number generation function (*rnorm*). Participants were assigned at random to either Track 1 or Track 2, which determined whether they completed the choking-involved or non-choking session first. Assignment was concealed from both participants and study staff to maintain blinding throughout the protocol.

Participants completed a baseline survey and then, throughout the study period, completed a brief daily questionnaire that was sent to them by text message each day. This 9-item survey captured details about recent sexual activity, including whether choking occurred, along with other contextual factors (e.g., instances of slapping during sex, menstrual cycle status) to help maintain blinding to the study's primary focus. When a qualifying event was reported (e.g., choking-involved sex or non-choking involved sex), study staff reached out to schedule a follow-up session within 24 h. Survey completion adherence was high, with an overall compliance rate of 94.7%.

#### Questionnaires

Participants completed an online baseline questionnaire that assessed their age, sex, race, ethnicity, concussion history, sexual history, and history of being choked/strangled during sex. Additionally, the following measures were administered. Depressive symptoms were measured with the Patient Health Questionnaire-9 (PHQ-9), a 9-item tool scored on a 4-point scale (0–3), with totals ranging 0–27 and higher scores reflecting greater severity (35). Stress was assessed using the Perceived Stress Scale (PSS), a 10-item measure rated on a 5-point scale (0–4), producing scores from 0 to 40, with higher scores indicating greater perceived stress (36). Anxiety was evaluated with the Generalized Anxiety Disorder-7 (GAD-7), a 7-item scale aligned with PHQ-9 scoring, yielding totals of 0–21, where higher values denote more severe anxiety (37). ADHD symptoms were assessed with the Adult ADHD Self-Report Scale (ASRS), which captures inattentive and hyperactive-impulsive traits in adults (38). Hazardous alcohol use was measured with the Alcohol Use Disorders Identification Test (AUDIT), a 10-item screening tool developed by the World Health Organization (39). Cannabis use was evaluated with the Cannabis Use Disorder Identification Test (CUDIT), which quantifies frequency and problematic use (40). Migraine burden was measured with the Migraine Disability Assessment Test (MIDAS), a 5-item scale assessing headache-related disability across work, household, and social domains (41).

#### Blood biomarker assessments

At each of the laboratory visit (baseline, choking-involved sex, non-choking sex), 6 mL of venous blood samples were collected on EDTA vacutainer sterile tubes and also on serum vacutainer sterile tubes with clot activator and silicone coat (BD Bioscience). Plasma was separated by centrifugation (1,500 x g, 15 min at 4 °C) and stored at −80 °C until analysis. Serum samples were allowed to clot for 30 min at room temperature and processed the same methods as the plasma samples. Brain-injury blood biomarkers, including GFAP, UCH-L1, tau, and NfL, were analyzed in plasma samples by the Human Neurology 4-Plex A assay with 1:4 dilution factor on a Quanterix SR-X system. The lowest detection limit of the assay was 0.771 pg/mL for GFAP, 12.0 pg/mL for UCH-L1, 0.112 pg/mL for tau, and 0.386 pg/mL for NfL. Another marker, S100B, was tested on serum samples instead of plasma due to its interaction with calcium ions within EDTA additives, using a sandwich-based enzyme-linked immunosorbent assay (ELISA) kit (S100B: EZHS100B-33 K, Millipore). The lowest detection limit of the assay was 2.7 pg/mL.

For inflammatory markers, Human Magnetic Luminex Assays were designed using the assay service from R&D Systems. The custom Luminex multiplex assays used a three-fold plasma sample dilution to quantify a panel of inflammatory cytokines (IL-1ra), inflammatory cytokine receptors (TNF-R1), and vascular integrity markers (VCAM-1), and used a two-fold plasma sample dilution to quantify a panel of chemokines (CCL-2) and vascular development markers (VEGF-A) in plasma samples. The lowest detection limits of the assay were 46.35 pg/mL for IL-1ra, 69.56 pg/mL for TNF-R1, 0.47 pg/mL for CCL-2, 1.35 pg/mL for VEGF-A, and 20.0 pg/mL for VCAM-1.

### Statistical analysis

Mixed-effects regression models (MRMs) were used to examine changes in brain injury blood biomarkers (GFAP, UCH-L1, NfL, tau, S100B) and systemic inflammatory markers (IL-1ra, TNF-R1, CCL-2, VEGF-A, and VCAM-1) across exposure types (post-choking and post-non-choking) relative to baseline. Each participant contributed data to both exposures in a repeated-measures design. Models included fixed effects for exposure, timepoint (baseline vs. post), and their interaction, with age and concussion history included as covariates. A random intercept and slope for timepoint was included to account for individual variability. Results were summarized using contrast estimates with its 95% confidence interval (CI) and *p*-values in the following format: [b estimate (95% CI, low CI-high CI), *p*-value]. Significance was set at *p* < 0.05. *post-hoc* analyses used marginal means to examine exposure differences as needed. All analyses were conducted using R (v4.5.1) using nlme and emmeans packages.

## Results

### Demographics

Of the 46 adult females screened, 43 met inclusion criteria and were enrolled. One participant withdrew prior to the baseline visit. After baseline, six were excluded from the study due to changes in sexual activity status, contraindications for other measures (e.g., MRI), or a neurological condition, and five withdrew due to scheduling conflicts. Two additional participants were excluded from blood draws at follow-up timepoints due to phlebotomy challenges during the baseline visit. After the first follow-up, one participant withdrew and three were excluded from the study due to changes in sexual activity. Final analyses included 29 participants (21.5 ± 2.67 years), randomized to Track 1 (*n* = 12) or Track 2 (*n* = 17). Sexual choking/strangulation events during the study were reported as consensual. See Figure 1 for the study flow. Demographic information is described in Table 1.

**Table 1:** Baseline demographics.

Variables	Participants
Sex, *n* (%)	Female, 29 (100)
Age, y	21.5 (2.67)
Race, *n* (%)	
White/Caucasian	21 (72.41)
Asian	3 (10.35)
Black or African American	1 (3.45)
Multi-race	3 (10.35)
Prefer Not to Say	1 (3.45)
Ethnicity, *n* (%)	
Not Latino/ Hispanic	26 (89.66)
Latino/ Hispanic	3 (10.35)
Education, *n* (%)	
High school diploma or GED	5 (17.24)
Some college, but no degree	13 (44.82)
Bachelor's Degree	9 (31.03)
Graduate or Professional Degree	2 (6.90)
No. of Previous Concussions, *n* (%)	
0	18 (62.07)
1	6 (20.69)
2	4 (13.79)
3+	1 (3.45)
Mental Health Diagnosis, *n* (%)	
No diagnosis	9 (31.03)
At least one diagnosis^a^	20 (68.97)
Depression	12 (41.38)
Anxiety	18 (62.07)
ADHD	5 (17.24)
PTSD	3 (10.35)
Use of Mental Health Related medication, *n* (%)	
Yes	11 (37.93)
No	18 (62.07)
Mental Health Symptoms, mean ± SD	
PHQ-9	5.82 (4.94)
GAD-7	5.68 (4.29)
PSS	18.18 (5.77)
ARSR	11.45 (3.64)
AUDIT	5.21 (3.34)
CUDIT	5.03 (5.38)
MIDAS	2.72 (4.76)

a Sum of individual mental health diagnosis exceeds 100% because of various combinations of multiple mental health diagnosis. ADHD, attention-deficit/hyperactivity disorder; PTSD, post-traumatic stress disorder; PHQ-9, patient health questionnaire; GAD-7, generalized anxiety disorder; PSS, perceived stress scale; ARSR, Adult ADHD Self-Report Scale; AUDIT, Alcohol Use Disorders Identification Test; CUDIT, Cannabis Use Disorders Identification Test; MIDAS, Migraine Disability Assessment.

### Brain-injury blood biomarkers

**Figure 2:**
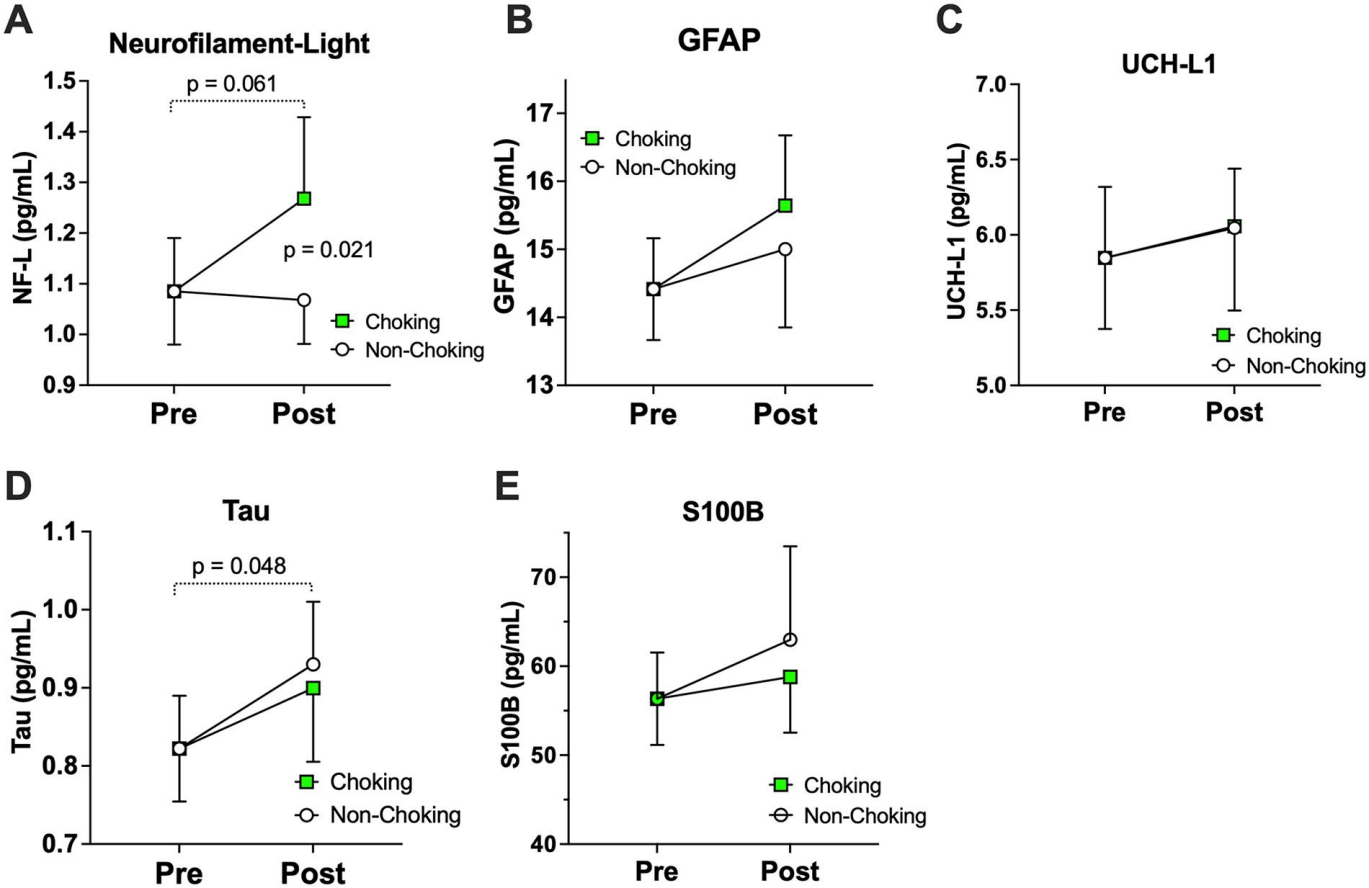
Event level differences in brain injury blood biomarkers. Significant exposure (choking vs. non-choking) by time (pre vs. post) interaction were found in NfL levels with the post-choking event exhibiting higher levels. There was no significant difference in the other brain-injury blood biomarkers. **(A)** Neurofilament-light, **(B)** GFAP, **(C)** UCH-L1, **(D)** Tau, and **(E)** S100B.

Most blood biomarkers of brain injury exhibited upward trends at the post-choking timepoint relative to baseline (Figure 2). Yet, NfL was the only marker to show a statistically significant differential response based on sexual event type. Specifically, NfL levels acutely increased following choking-involved sex [*b* = 0.17 (−0.01, 0.34), *p* = 0.061] but remained unchanged after non-choking sex [*b* = −0.04 (−0.22, 0.14), *p* = 0.657; Table 2]. This pattern yielded a statistically significant exposure (choking vs. non-choking) by time (pre vs. post) interaction [*b* = −0.21 (−0.38, −0.03), *p* = 0.021]. None of the other 4 markers (GFAP, tau, UCH-L1, S100B) differed in terms of statistical significance between choking and non-choking sex.

**Table 2:** Changes in blood biomarker levels compared to baseline. Group difference derived from exposure by time interactions.

Types of biomarkers	Biomarkers	Post-choking	Post-non-choking	Group difference
Brain Injury Biomarkers	GFAP	0.91	0.90	−0.01
		[−0.20, 2.03]	[−0.22, 2.03]	[−0.95, 0.92]
		0.105	0.114	0.977
	NfL	0.17	−0.04	−0.21
		[−0.01, 0.34]	[−0.22, 0.14]	[−0.38, −0.03]
		0.061	0.657	0.021
	UCH-L1	0.19	0.14	−0.04
		[−0.97, 1.34]	[−1.02, 1.31]	[−0.91, 0.82]
		0.745	0.806	0.919
	Tau	0.07	0.14	0.08
		[−0.07, 0.20]	[0.001, 0.28]	[−0.03, 0.18]
		0.347	0.048	0.150
	S100B	2.49	4.48	1.99
		[−7.58, 12.60]	[−5.84, 14.80]	[−7.01, 10.99]
		0.622	0.388	0.659
Inflammatory Biomarkers	VCAM−1	−18,159.0	−37,250.0	−19,091.0
		[−92,249, 55,931]	[−1,11,790, 37,290]	[−70,790, 32,608]
		0.624	0.319	0.462
	TNF-R1	−17.30	−13.50	3.81
		[−67.90, 33.20]	[−64.50, 37.50]	[−35.80, 43.50]
		0.495	0.597	0.848
	CCL−2	16.85	2.29	−14.60
		[1.50, 32.20]	[−13.20, 17.70]	[−25.70, −3.43]
		0.032	0.767	0.011
	VEGF-A	5.17	−4.12	−9.29
		[−6.20, 16.53]	[−15.60, 7.37]	[−19.71, 1.13]
		0.367	0.476	0.079
	IL−1ra	−18.80	−40.80	−21.90
		[−166.0, 128.0]	[−188.0, 106.0]	[−81.90, 38.00]
		0.796	0.577	0.467

Values expressed as Beta-value [95% CI], *p*-value.

### Systemic inflammatory markers

**Figure 3:**
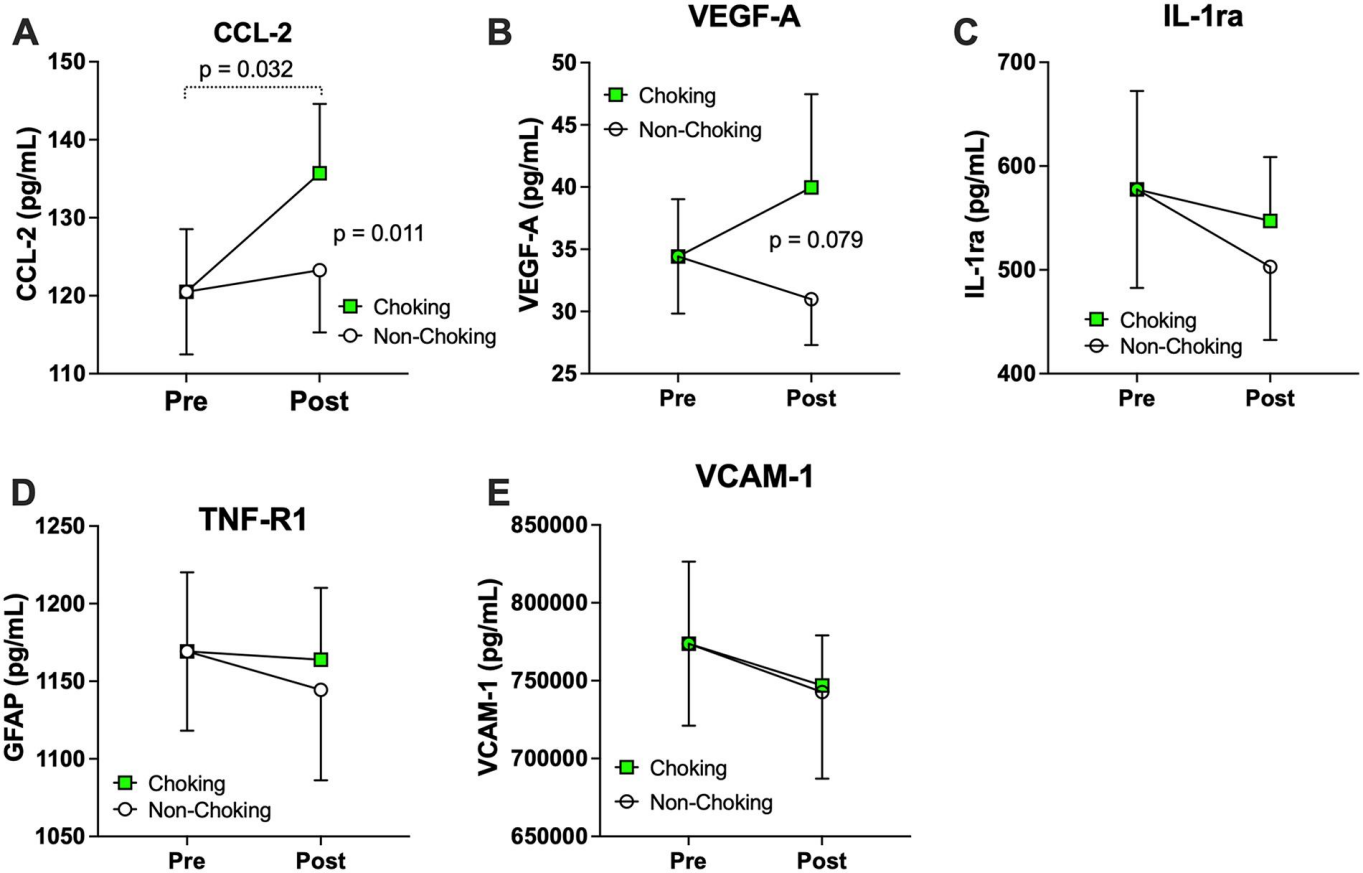
Systemic markers exhibiting event level differences. Significant group differences were found in CCL2 levels with the post choking group exhibiting higher levels in comparison to baseline. There was no comparable change seen in CCL2 levels following non-choking sex, resulting in a significant exposure-by-time interaction. VEGF-A demonstrated a similar pattern, albeit not significantly, resulting in a marginal exposure-by-time interaction with the choking group showing higher levels. There was no significant difference in the other systemic blood biomarkers tested. **(A)** CCL-2, **(B)** VEGF-A, **(C)** IL-1ra, **(D)** TNF-R1, and **(E)** VCAM-1.

Several systemic inflammatory and vascular markers demonstrated differential changes between choking- and non-choking–involved sexual events. Notably, CCL-2 increased significantly at post-choking sex compared to baseline [*b* = 16.85 (1.50, 32.20), *p* = 0.032], whereas there was no comparable change following non-choking sex; this resulted in a significant exposure-by-time interaction (*b* = −14.60 [−25.70, −3.43, *p* = 0.011). While not statistically significant, VEGF-A exhibited a similar pattern, showing an acute post-choking increase [*b* = 5.17 (−6.20, 16.53), *p* = 0.367] relative to baseline that contrasted with a decline after non-choking sex [*b* = −4.12 (−15.60, 7.37), *p* = 0.476], resulting in a marginal exposure-by-time interaction [*b* = −9.29 (−19.71, 1.13), *p* = 0.079: Figure 3]. In contrast, IL-1ra, TNF-R1, and VCAM-1 remain consistent regardless of choking or non-choking event (Table 2).

## Discussion

This randomized crossover study is, to our knowledge, the first to investigate acute neurobiological responses to consensual sexual choking/strangulation, and more broadly, to any form of strangulation, in young adult women. This study yielded several notable findings. First, among the five brain-injury biomarkers assessed, NfL was the only marker to demonstrate a significant exposure-by-time interaction, showing an acute increase after choking-involved sex but no change after non-choking sex, relative to baseline. Second, CCL-2, which is a chemokine involved in monocyte recruitment and BBB regulation (42), rose significantly post-choking with no comparable change post-non-choking relative to baseline, indicating a selective inflammatory response to ischemic/hypoxic stress. VEGF-A, a protein that plays a crucial role in angiogenesis (43), also exhibited a similar pattern as CCL-2. Lastly, other inflammatory mediators along vascular and neurotrophic markers were unaffected, among females in this sample, by acute exposure to sexual choking/strangulation. Collectively, our data indicate that even in consensual contexts, sexual choking/strangulation can elicit transient changes in select blood biomarkers indicative of axonal stress and inflammation, suggesting that even brief episodes of neck compression during sex may be biologically deleterious. Findings from our brain injury biomarkers provide novel insights into the acute neural consequences of sexual choking/strangulation. Particularly, plasma NfL demonstrated a differential response, with levels increasing following choking-involved sex but remaining stable after non-choking sex. NfL is widely recognized as one of the most sensitive biomarkers for detecting subtle and even asymptomatic neuronal stress (27, 44, 45), as well as for its diagnostic and prognostic utility in the subacute and chronic stages of brain injury (46, 47). In contrast, the combined measurement of GFAP and UCH-L1 is suited for detecting acute injury and the presence of intracranial hemorrhage within the first several hours post-trauma (48, 49). Because our sampling occurred primarily 12–24 h post-exposure, the superior performance of NfL over astroglial or neuronal markers is consistent with the temporal dynamics established in biomarker literature (50). Mechanistically, strangulation differs from mechanical injuries such as concussions or head impacts. Rather than direct structural disruption to the brain, strangulation induces hypoxic–ischemic stress that initiates molecular cascades leading to axonal destabilization. These cascades are mediated by processes such as calcium overload, cytoskeletal degradation, and impaired axonal transport (51). NfL is particularly sensitive to this pathophysiology due to its enrichment in myelinated axons within deep white matter tracts, which are highly dependent on uninterrupted cerebral blood flow (52). Consequently, even brief hypoxic episodes can destabilize axonal cytoskeletal integrity without gross neuronal death.

Our findings should be interpreted in relation to the prior study examining blood biomarkers in women with sexual choking/strangulation exposure. Huibregtse et al. (28) reported elevated S100B, but not NfL, among young women with recent and frequent partnered strangulation. The prior study examined exposure history rather than acute events and assessed biomarkers at variable timepoints that did not consistently fall within a defined post-exposure window that we employed in this study (∼24 h). Our randomized crossover design focuses on acute (<24 h) responses. The lack of S100B elevation in the present study may reflect the timing sensitivity of S100B and GFAP, which peak early (1–6 h) and may normalize before our sampling window. Conversely, NfL rises more slowly and remains elevated for days to weeks after axonal stress, which aligns with our observed acute response. These methodological differences, including acute vs. cumulative exposure, timing of sampling, and crossover vs. observational designs, may explain divergent biomarker patterns.

Our inflammatory panel revealed a selective pattern. CCL-2 increased within 24 h after choking-involved sex, and VEGF-A trended upward in the same direction, whereas IL-R1, TNF-R1, and VCAM-1 did not differ by exposure. This profile aligns with a hypoxia-ischemia-related mechanism rather than a broad systemic cytokine surge. CCL-2 is a principal monocyte chemoattractant induced by hypoxia and endothelial stress. Its upregulation can promote perivascular immune cell recruitment and modulate BBB function (42). Oxygen deprivation can trigger CCL-2 upregulation through hypoxia inducible factor-1alpha (HIF-1α), which triggers neuroinflammatory cascades and prime monocyte recruitment and microglial activation in brain tissues (53). Furthermore, preclinical data by Joly-Amado et al. (54) suggests that an elevation of CCL-2 can trigger astroglial activation and accelerate tau protein accumulation in mice brain, pointing to the role of CCL-2 in neurodegenerative conditions. VEGF-A, likewise regulated by hypoxia pathways, orchestrates angiogenesis and vascular permeability (43). In acute settings, transient VEGF-A increases may be adaptive by restoring perfusion but can also loosen tight junctions of the BBB and increase risks for edema formation (55). A similar pattern observed between CCL-2 and VEGF-A after sexual choking/strangulation supports clinical observations that non-fatal strangulation is accompanied by inflammatory signatures and microvascular compromise, even when gross imaging (e.g., CT scan) shows negative results (56).

By contrast, we did not detect exposure-specific differences for IL-1ra, TNF-R1, or VCAM-1. Two study design features likely contributed. First, cytokines, such as IL-6 and TNF-α and antagonists like IL-1ra, typically peak within one to three hours of an inflammatory trigger and can normalize rapidly, whereas adhesion molecules and some receptors can show delayed or context-dependent kinetics (32). Sampling primarily at 12–24 h post-event may therefore miss early cytokine peaks. This is consistent with meta-analysis data in mild TBI, which show significant IL-6/IL-1ra elevations within the first 24 h; most robust at earlier timepoints (57). Second, consensual non-choking sexual activity itself can induce transient systemic changes (e.g., hemodynamic and endocrine shifts) that may obscure small exposure-specific effects unless they are strongly hypoxia-linked, as appears to be the case for CCL-2/VEGF-A. Overall, our findings suggest that consensual sexual choking/strangulation elicits a specific inflammatory response (CCL-2 and/or VEGF-A) linked to hypoxia rather than a broad cytokine surge. This complements prior IPV work showing strangulation-related inflammatory profiles and neurobehavioral impairment (34) and extends a broader neurotrauma literature indicating that inflammatory and endothelial signaling can be activated in the absence of overt structural injury.

To date, there is no scientifically established “safe duration” of neck compression in the contexts of sexual choking/strangulation, IPV strangulation, autoerotic hypoxia, or self-asphyxia behaviors (17). Preclinical models indicate that even 15–30 s of cerebral hypoxia can initiate calcium-mediated cytoskeletal degradation and inflammatory signaling (34, 51), and clinical reports document neurological sequelae after similar brief exposures (19, 58). The absence of normative data underscores the need for research on exposure dose–response patterns. Although the clinical significance of these acute biomarker elevations remains uncertain, elevations in NfL and CCL-2 in other hypoxic or neurotrauma contexts have been associated with axonal stress (26, 27, 59, 60), endothelial dysfunction, and microvascular permeability (33, 54). We emphasize that our findings do not imply neurological injury *per se*, but rather biological responses consistent with transient ischemic stress. Longitudinal studies integrating cumulative exposure history, neuroimaging, and behavioral outcomes will be required to clarify short- and long-term consequences.

### Limitations

There are several limitations to this study. First, the sample size was modest and restricted to young adult women, which hinders generalizability to men, gender-diverse individuals, and other age cohorts. Nonetheless, this work represents an important first step in providing foundational knowledge for future investigations across diverse populations. Second, exposure to sexual choking/strangulation was based on self-report. Misclassification of exposure intensity and unmeasured variability in technique (e.g., pressure on the carotid arteries vs. the trachea; amount of pressure; etc.) may have introduced noise, potentially biasing results toward the null. Third, blood samples were obtained primarily 12–24 h post-event. Many cytokines and endothelial signals peak within the first few hours and normalize quickly, whereas some neural-injury biomarkers rise more slowly. As such, our timing may have missed early pro-inflammatory peaks and the later trajectory of axonal markers. Repeated sampling at multiple intervals would better capture the temporal response profiles of neuronal and inflammatory markers. Although participants reported choking intensity, number of compressions, and mode of application during post-choking visit, we elected not to include these data in the present biomarker-focused analysis. Subjective choking intensity lacks a validated or widely accepted measurement scale, shows high within-person variability, and may not reliably correspond to physiological load or vascular compression patterns. Future studies should conduct validation, standardization, and reliability assessments on these exposure characteristics. Finally, the clinical implications of these biomarker responses remain to be determined. Future studies should integrate complementary measures, such as functional MRI, cerebrovascular reactivity tests, and behavioral outcomes (e.g., eye-tracking), to clarify the neurobiological and functional significance of these findings.

## Conclusions

This randomized crossover study provides the first evidence that consensual sexual strangulation/choking elicits acute neurobiological responses detectable in peripheral blood. We observed a selective increase in NfL, a highly sensitive marker of axonal stress, and a concurrent rise in CCL-2, with VEGF-A trending in the same direction. These data indicate that brief hypoxic–ischemic episodes can activate axonal injury cascades and hypoxia-responsive inflammatory pathways. While preliminary, our results highlight the potential utility of blood-based biomarkers for capturing subtle neural stress associated with sexual choking/strangulation and provide a foundation for future studies incorporating multimodal biomarkers, neuroimaging, and clinical outcomes. Ultimately, elucidating the acute and cumulative consequences of sexual strangulation is essential to inform education, risk assessment, and clinical care in both consensual and non-consensual contexts. Future work should integrate biological measures with psychological, relational, and sexual-satisfaction outcomes to support a more comprehensive understanding of both the risks and subjective benefits associated with this behavior, thus informing education and clinical decision-making.

## Data Availability

The raw data supporting the conclusions of this article will be made available by the authors, without undue reservation.
